# Synthesis and Anticancer Activity of Some New Pyrazolo[3,4-*d*]pyrimidin-4-one Derivatives

**DOI:** 10.3390/molecules19033297

**Published:** 2014-03-18

**Authors:** Khaled R. A. Abdellatif, Eman K. A. Abdelall, Mohamed A. Abdelgawad, Rasha R. Ahmed, Rania B. Bakr

**Affiliations:** 1 Pharmaceutical Organic Chemistry Department, Faculty of Pharmacy, Beni-Suef University, Beni-Suef 62514, Egypt; E-Mails: khaled.ahmed@bsu.edu.eg (K.R.A.A.); Emansherif94@yahoo.com (E.K.A.A.); mhmdgwd@yahoo.com (M.A.A.); 2 Cell Biology and Histology Division, Zoology Department, Faculty of Science, Beni-Suef University, Beni-Suef 62514, Egypt; E-Mail: shorouk2002os@yahoo.com

**Keywords:** pyrazolo[3,4-*d*]pyrimidin-4-one, anticancer activity, MCF7

## Abstract

3,6-Dimethyl-1-phenyl-1*H*-pyrazolo[3,4-*d*][1,3]oxazin-4-one (**3**) was prepared by hydrolysis of ethyl 5-amino-3-methyl-1-phenyl-1*H*-pyrazole-4-carboxylate (**1**) to afford the corresponding carboxylic acid **2**, which was reacted with acetic anhydride to give **3**. The pyrazolo[3,4-*d*][1,3]oxazin-4-one **3** was reacted with hydroxylamine hydrochloride, urea, thiourea, thiosemicarbazide, phenylhydrazine and aromatic amines to afford the corresponding pyrazolo[3,4-*d*]pyrimidin-4-ones **4**, **5a**,**b**, **6**, **7**, **8a**–**e**, respectively. Condensation of pyrazoloxazine derivative **3** with 99% hydrazine hydrate afforded the 5-aminopyrazolo[3,4-*d*]pyrimidine derivative **9**. Coupling of **9** with aromatic aldehydes yielded a series of 3,6-dimethyl-5-(4-substitutedbenzylideneamino)-1-phenyl-1,5-dihydropyrazolo[3,4-*d*]pyrimidin-4-ones **10a**–**e**. The new compounds were tested for their antitumor activity on the MCF-7 human breast adenocarcinoma cell line. Almost all the tested compounds revealed antitumor activity, especially 3,6-dimethyl-5-(4-nitrobenzylideneamino)-1-phenyl-1,5-dihydropyrazolo[3,4-*d*]pyrimidin-4-one (**10e**) which displayed the most potent inhibitory activity with a half maximal inhibitory concentration (IC_50_) of 11 µM.

## 1. Introduction

Cancer remains one of the most life-threatening diseases, taking nearly 7 million lives each year worldwide. It is realized that neither surgery nor radiation nor the two in combination can adequately control metastatic cancer [1], therefore, efforts to cure cancer have been focusing on conventional chemotherapy. However, this type of treatment usually does not discriminate between dividing normal cells and tumor cells, leading to severe side effects [2]. In the last decade, the use of molecular targeted therapies (a new generation of selective cancer drugs which interfere with specific receptors and signaling pathways that promote tumor cell growth) has made treatments more tumor-specific [3].

The chemistry of pyrazolo[3,4-*d*]pyrimidine derivatives has received great attention due to their structural similarity with purines and hence several pyrazolo[3,4-*d*]pyrimidine derivatives exhibit promising anticancer activity [4,5,6,7,8,9]. Different mechanisms account for the cytotoxic effect of this class of compounds, where they had been reported to act as glycogen synthase kinase (GSK) inhibitors [10], cyclin dependent kinase (CDK) inhibitors [11], dual src/Ab1 kinase inhibitors [12] and epidermal growth factor receptor (EGFR) inhibitors [13]. Moreover, many 5-substituted-1-phenyl-1*H*-pyrazolo[3,4-*d*]pyrimidin-4-ones were reported to possess antiproliferative activity against breast carcinoma, MCF7 [14,15].

Examples of anticancer drugs currently used in anticancer therapy can be represented by erlotinib (Tarceva^TM^) [16] and gefitinib (Iressa^TM^) [17] which have been approved for the chemotherapeutic treatment of patients with advanced non-small lung cancer. Also, lapatinib (Tykerb^TM^) [18] was approved for the treatment of breast cancer.

**Figure 1:**
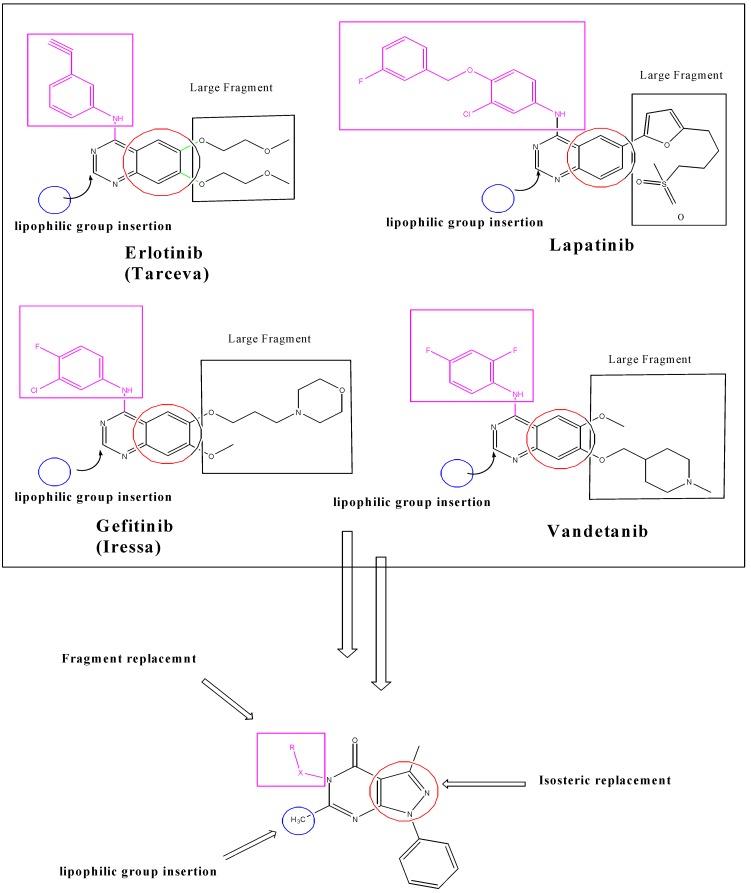
Planned design of new pyrazolo[3,4-*d*]pyrimidine derivatives for cytotoxic activity.

In the view of the previous rationale and in continuation of an ongoing program on the synthesis of antitumor compounds [19], in the present study a new series of pyrazolo[3,4-*d*]pyrimidin-4-ones has been synthesized and screened *in vitro* for antitumor activity. The series comprises the derived 5,6-disubstituted pyrazolo[3,4-*d*]pyrimidin-4-one pharmacophore that is structurally related to erlotinib and lapatinib (Figure 1). In the present study, the substitution pattern at the 5,6-disubstituted pyrazolo[3,4-*d*]pyrimidin-4-one pharmacophore was manipulated so as to create different electronic environments that might affect the lipophilicity and hence the activity of target molecules.

The rationale for the design of target compounds was based upon some structural modifications on the general features of anilinoquinazoline-containing compounds (Figure 1). These modifications comprise a replacement of the benzene moiety in the quinazoline skeleton by a pyrazolo moiety as the pyrazolo moiety is naturally found in the body’s purine bases and this is expected to enhance cytotoxic activity. Prompted by these claims, we present a new series of compounds containing 5,6-disubstituted pyrazolo[3,4-*d*]pyrimidin-4-ones core as anticancer agents. Our strategy is directed toward designing a variety of compounds with diverse chemical properties hypothesizing that the potency of these compounds might be increased by adding alternative binding groups such as a methyl group at position 6, and aroylhydrazone, phenylamino, amide, thioamide, thiosemicarbazide and substituted aryl at position 5 of the pyrazolo[3,4-*d*]pyrimidine ring.

## 2. Results and Discussion

### 2.1. Chemical Synthesis

**Scheme 1:**
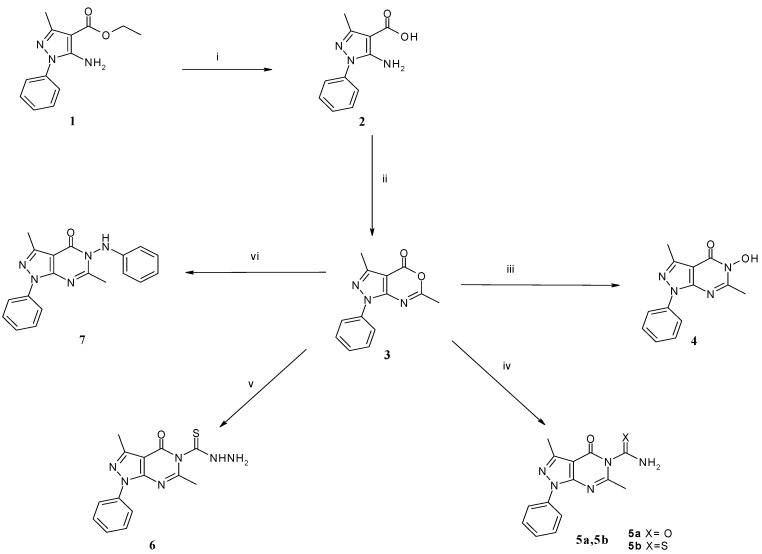
Synthetic pathway for target compounds **4**, **5a**,**b**, **6**, **7**.

**Scheme 2:**
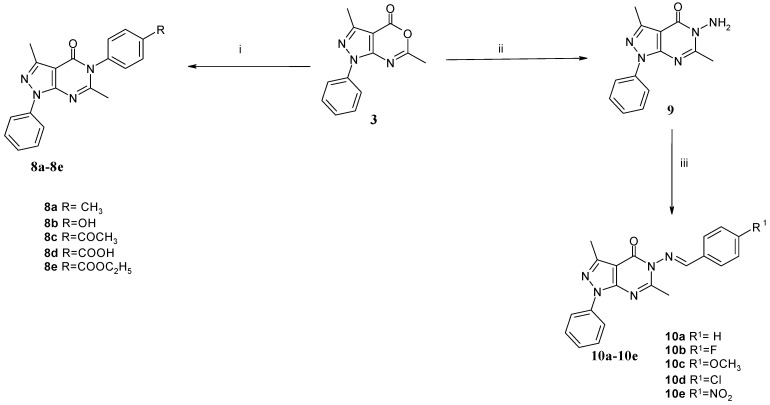
Synthetic pathway for target compounds **8a**–**e** and **10a**–**e**.

The synthesis of the target compounds is outlined in Scheme 1 and Scheme 2. Accordingly, basic hydrolysis of ethyl 5-amino-3-methyl-1-phenyl-1*H*-pyrazole-4-carboxylate (**1**) [20] gave 5-amino-3-methyl-1-phenyl-1*H*-pyrazole-4-carboxylic acid (**2**) (Scheme 1). The formation of compound **2** was confirmed by ^1^H-NMR that showed two D_2_O exchangeable singlet signals at δ 6.30, 12.08 ppm corresponding to NH_2_ and COOH, respectively. Heating compound **2** with acetic anhydride gave a cyclized product, 3,6-dimethyl-1-phenyl-1*H*-pyrazolo[3,4-*d*][1,3]oxazin-4-one (**3**). The ^1^H-NMR spectrum of compound **3** revealed the appearance of a singlet signal at δ 2.49 ppm corresponding to the pyrimidine CH_3_ protons. The mass spectrum of compound **3** showed a molecular ion peak at *m/z* 241 which appeared as the base peak. Reaction of compound **3** with hydroxylamine hydrochloride in dry pyridine afforded 5-hydroxy-3,6-dimethyl-1-phenyl-1,5-dihydropyrazolo[3,4-*d*]pyrimidin-4-one (**4**). The presence of a singlet OH band at 3,426 cm^−1^ and C=O at 1,680 cm^−1^ confirmed the formation of **4**. Its ^1^H-NMR spectrum showed the appearance of a D_2_O exchangeable singlet signal at δ 11.52 ppm corresponding to the OH proton. The target compounds **5a**,**b** and **6** were synthesized by fusion at 200 °C of pyrazoloxazine derivative **3** with urea, thiourea and thiosemicarbazide, respectively. The formation of compounds **5a**,**b** and **6** was confirmed by their ^1^H-NMR spectra that indicated the appearance of exchangeable singlet signals at δ 11.15–12.45 ppm corresponding to NH and NH_2_. In addition, the mass spectra agreed with the calculated molecular weights of the expected products. On the other hand, reaction of pyrazoloxazine derivative **3** with phenylhydrazine afforded 3,6-dimethyl-1-phenyl-5-phenylamino-1,5-dihydropyrazolo[3,4-*d*]pyrimidin-4-one (**7**). The ^1^H-NMR of this compound revealed in addition to the corresponding integration for aromatic protons, the presence of NH at δ 9.09 ppm. The mass spectrum also showed a molecular ion peak at *m/z* 331 as the base peak.

*Reagents and conditions*: (i) NaOH, CH_3_OH, reflux 5 h; (ii) (CH_3_CO)_2_O, reflux 5 h; (iii) NH_2_OH·HCl, dry pyridine, reflux 8h; (iv) NH_2_CXNH_2_, fusion at 200 °C 1 h; (v) NH_2_CSNHNH_2_, fusion at 200 °C 1 h; (vi) RC_6_H_4_NH_2_, dry pyridine, reflux 6 h.

*Reagents and conditions*: (i) RC_6_H_4_NH_2_, dry pyridine, reflux 6 h; (ii) NH_2_NH_2_·H_2_O, *n*-butanol, reflux 6 h; (iii) RC_6_H_4_CHO, acetic acid, reflux 20 h.

Moreover, reaction of compound **3** with appropriate aromatic amines furnished 3,6-dimethyl-1-phenyl-5-(4-substitutedphenyl)-1,5-dihydropyrazolo[3,4-*d*]pyrimidin-4-ones **8a**–**e** (Scheme 2). The structure of these compounds was established on the basis of their elemental analyses and spectral data. The ^1^H-NMR spectra revealed signals at δ 6.89-8.15 ppm for aromatic protons. Also, the mass spectra were in agreement with the calculated molecular weights of the synthesized compounds. Condensation of pyrazoloxazine derivative **3** with hydrazine hydrate afforded the 5-amino-pyrazolo[3,4-*d*]pyrimidin-4-one derivative **9**. Finally, coupling of **9** with appropriate aromatic aldehydes yielded the corresponding 5-(substituted benzylideneamino)-pyrazolo[3,4-*d*]pyrimidine derivatives **10a**‒**e**. The structures of **10a**–**e** were established on the basis of their elemental analysis and spectral data. The ^1^H-NMR spectra of **10a**–**e** showed singlet downfield CH=N signals at δ 8.32–10.94 ppm. It is worth mentioning that the lack of NH_2_ bands in the IR spectra and D_2_O exchangeable NH_2_ signals in the ^1^H-NMR spectra confirmed the production of the pyrazolo[3,4-*d*]pyrimidine derivatives **10a**–**e**.

### 2.2. In Vitro Anticancer Screening

The newly synthesized compounds were evaluated for their *in vitro* cytotoxic activity against human breast cell line (MCF7) using doxorubicin as the reference drug according to the method described as reported by Vichai and Kirtikara [21]. The cytotoxicity was assessed at concentrations of 0, 0.01, 0.1, 10 and 100 µg/mL. The relation between surviving fraction and drug concentration was plotted to obtain the survival curve of MCF7 tumor cell line after addition of the specified compound. The parameter used here is IC_50_, which corresponds to the concentration required for 50% inhibition of cell viability. The IC_50_ values of the synthesized compounds compared to the reference drug are shown in Table 1.

**Table 1:** Results of *in vitro* cytotoxic activity of the synthesized compounds on human breast cancer cell line (MCF7).

Compound No.	IC_50_ in µM
Doxorubicin	5
**4**	49
**5a**	52
**5b**	38
**6**	52
**7**	14
**8a**	33
**8b**	25
**8c**	26
**8d**	25
**8e**	27
**9**	84
**10a**	17
**10b**	12
**10c**	18
**10d**	12
**10e**	11

The obtained data revealed that most of the newly synthesized compounds showed potent antitumor activity. Among the tested compounds, the most potent cytotoxic effect against MCF-7 cell line was obtained with the compound 5-(4-nitrobenzylideneamino)pyrazolo[3,4-*d*]pyrimidin-4-one (**10e**) with an IC_50_ value of 11 µM, followed by **10d** which showed an IC_50_ value of 12 µM. Compound **9** exhibited the least cytotoxic activity.

From the antitumor screening results the against the MCF-7 cell line, some structure activity relationships can be suggested. Aromatic substitution on N5 position favors the activity; this is obvious upon comparing 5-(4-hydroxyphenylpyrazolo[3,4-*d*]pyrimidin-4-one derivative **8b** (IC_50_ = 25) and 5-hydroxy-3,6-dimethyl-1-phenyl-1,5-dihydropyrazolo[3,4-*d*]pyrimidin-4-one (**4**) (IC_50_ = 49). Also, the introduction of a 5-amino function on the pyrazolo[3,4-*d*]pyrimidine core produced inactive compound **9** (IC_50_ = 84), while the introduction of an anilino function favors the anticancer activity as shown by compound **7** (IC_50_ = 14). Conversion of inactive compound **9** into a 5-substituted benzylidene amino function yielded compounds **10a**–**e**, the most active members of this study. The influence of substituents on the benzylideneamino group on the antitumor activity was in the order NO_2_ > F > Cl > OCH_3_ > H. Moreover, 5-(4-substituted benzylideneamino) derivatives **10a**–**e** with azomethine spacer groups were more potent than the 5-(4-substituted phenyl) derivatives **8a**–**e**.

## 3. Experimental

### 3.1. General

Melting points were determined on a Griffin apparatus and are uncorrected. IR spectra were determined on Shimadzu IR 435 spectrophotometer and values are presented in cm^−1^. ^1^H-NMR spectra were recorded on Varian Gemini 300 MHz spectrometer, using TMS as internal standard and chemical shifts are reported in ppm on the δ scale. The electron impact (EI) mass spectra were recorded on a Shimadzu QP-2010 plus instrument. Analytical thin layer chromatography (TLC) on silica gel plates containing UV indicator was routinely employed to follow the course of reactions and to check the purity of products. All reagents and solvents were purified and dried by standard techniques. Elemental microanalyses were carried out at the Microanalytical Center, Cairo University.

*5-Amino-3-methyl-1-phenyl-1H-pyrazole-4-carboxylic acid* (**2**). A mixture of ethyl 5-amino-3-methyl-1-phenyl-1*H*-pyrazole-4-carboxylate (**1**, 12.25 g, 50 mmol) and sodium hydroxide (4.20 g, 10 mmol) in methanol (60 mL) was heated under reflux for 5 h. After cooling, the reaction mixture was poured into ice-cold water, then adjusting pH of the mixture to 4 using concentrated hydrochloric acid. The solid obtained was filtered, dried and crystallized from ethanol/water. Mp 156–157 °C; yield: 56%; IR (cm^−1^): 3389–3204 (OH & NH_2_); 3009 (CH aromatic); 2927 (CH aliphatic); 1651 (C=O); 1613 (C=N); ^1^H-NMR (DMSO-*d_6_*) *δ* ppm: 2.25 (s, 3H); 6.30 (s, 2H); 7.36–7.54 (m, 5H); 12.08 (s, 1H); MS *m/z*: 217 (M^+^, 27.65);. Anal. Calcd for C_11_H_11_N_3_O_2_ (217.23): C, 60.82; H, 5.10; N, 19.34. Found: C, 60.69; H, 5.20; N, 19.67.

*3,6-Dimethyl-1-phenyl-1H-pyrazolo[3,4-d][1,3]oxazin-4-one* (**3**). A mixture of 5-amino-3-methyl-1-phenyl-1*H*-pyrazole-4-carboxylic acid (**2**, 2.17 g, 10 mmol) and acetic anhydride (5 mL) was heated under reflux for 5 h. After cooling, the formed solid was filtered, dried and crystallized from methanol. Mp 129–130 °C; yield: 49%; IR (cm^−1^): 3078 (CH aromatic); 2923 (CH aliphatic); 1764 (C=O); 1599 (C=N); ^1^H-NMR (DMSO-*d_6_*) *δ* ppm: 2.46 (s, 3H); 2.49 (s, 3H); 7.43 (t, *J* = 9 Hz, 1H); 7.57 (t, *J* = 9 Hz, 2H); 7.90 (d, *J* = 9 Hz, 2H); MS *m/z*: 241 (M^+^, 100). Anal. Calcd for C_13_H_11_N_3_O_2_ (241.25): C, 64.72; H, 4.60; N, 17.42. Found: C, 65.01; H, 4.73; N, 17.19.

*3,6-Dimethyl-5-hydroxy-1-phenyl-1,5-dihydropyrazolo[3,4-d]pyrimidin-4-one* (**4**). A mixture of compound **3** (2.41 g, 10 mmol) and hydroxylamine hydrochloride (0.69 g, 10 mmol) in dry pyridine (30 mL) was heated under reflux for 8 h. The reaction mixture was concentrated to its half volume and the separated solid was filtered, washed with water, dried and crystallized from dioxane. Mp 180–181 °C; yield: 74%; IR (cm^−1^): 3426 (OH); 2926 (CH aliphatic); 1680 (C=O); 1568 (C=N); ^1^H-NMR (DMSO-*d_6_*) *δ* ppm: 2.48 (s, 3H); 2.54 (s, 3H); 7.35 (t, *J* = 8.4 Hz, 1H); 7.53 (t, *J* = 8.4 Hz, 2H); 8.03 (d, *J* = 9 Hz, 2H); 11.52 (s, 1H); ^13^C-NMR (DMSO-*d_6_*) *δ* ppm: 13.71, 21.38, 105.07, 121.58, 126.92, 129.59, 138.75, 145.98, 149.94, 155.24, 157.86. MS *m/z*: 256 (M^+^, 89.42); Anal. Calcd for C_13_H_12_N_4_O_2_ (256.27): C, 60.93; H, 4.72; N, 21.86. Found: C, 60.81; H, 5.00; N, 21.66.

#### 3.1.1. General Procedure for the Synthesis of 3,6-Dimethyl-1-phenyl-5-substituted-1,5-dihydro-pyrazolo[3,4-*d*]pyrimidin-4-ones **5a**,**b**

A mixture of 3,6-dimethyl-1-phenyl-1*H*-pyrazolo[3,4-*d*][1,3]oxazin-4-one (**3**, 2.41 g, 10 mmol) and urea or thiourea (10 mmol) was fused at 200 °C for 1 h. The mixture was cooled and methanol was added to the reaction mixture. The separated solid was filtered, washed with methanol, dried and crystallized from ethanol.

*3,6-Dimethyl-4-oxo-1-phenyl-1,4-dihydropyrazolo[3,4-d]pyrimidine-5-carboxamide* (**5a**). Mp > 300 °C; yield: 65%; IR (cm^−1^): 3432–3344 (NH_2_); 3073 (CH aromatic); 2926 (CH aliphatic); 1671 (broad band of 2C=O); 1592 (C=N); ^1^H-NMR (DMSO-*d_6_*) *δ* ppm: 2.35 (s, 3H); 2.46 (s, 3H); 7.34 (t, *J* = 7.5 Hz, 1H); 7.51 (t, *J* = 7.5 Hz, 2H); 8.02 (d, *J* = 9 Hz, 2H); 12.20 (s, 2H); MS *m/z*: 283 (M^+^, 12.81); Anal. Calcd for C_14_H_13_N_5_O_2_ (283.29): C, 59.36; H, 4.63; N, 24.72. Found: C, 59.55; H, 4.52; N, 24.94.

*3,6-Dimethyl-4-oxo-1-phenyl-1,4-dihydropyrazolo[3,4-d]pyrimidine-5-carbothioamide* (**5b**). Mp > 300 °C; yield: 45%; IR (cm^−1^): 3425 (NH_2_); 2923 (CH aliphatic); 1675 (C=O); 1596 (C=N); 1118 (C=S); ^1^H-NMR (DMSO-*d_6_*) *δ* ppm: 2.38 (s, 3H); 2.48 (s, 3H); 7.35 (t, *J* = 7.2 Hz, 1H); 7.51 (t, *J* = 7.2 Hz, 2H); 8.02 (d, *J* = 9 Hz, 2H); 12.45 (s, 2H); Anal. Calcd for C_14_H_13_N_5_OS (299.36): C, 56.17; H, 4.38; N, 23.39. Found: C, 56.22; H, 4.21; N, 23.64.

*3,6-Dimethyl-4-oxo-1-phenyl-1,4-dihydropyrazolo[3,4-d]pyrimidine-5-carbothioic acid hydrazide* (**6**). A mixture of 3,6-dimethyl-1-phenyl-1*H*-pyrazolo[3,4-*d*][1,3]oxazin-4-one (**3**, 2.41 gm, 10 mmol) and thiosemicarbazide (0.91 g, 10 mmol) was fused at 200 °C for 1 h. The reaction mixture was cooled and methanol was added to the reaction mixture. The separated solid was filtered, washed with methanol, dried and crystallized from acetone. Mp > 250–251 °C; yield: 63%; IR (cm^−1^): 3321 (broad band of NH &NH_2_); 3064 (CH aromatic); 2920 (CH aliphatic); 1694 (C=O); 1590 (C=N); 1105 (C=S); ^1^H-NMR (DMSO-*d_6_*) *δ* ppm: 2.37 (s, 3H); 2.49 (s, 3H); 7.35 (t, *J* = 6 Hz,1H); 7. 51 (t, *J* = 7.2 Hz, 2H); 8.01 (d, *J* = 9 Hz, 2H); 11.15 (s, 1H); 12.19 (s, 2H); MS *m/z*: 314 (M^+^, 6.92); Anal. Calcd for C_14_H_14_N_6_OS (314.37): C, 53.49; H, 4.49; N, 26.73. Found: C, 53.28; H, 4.47; N, 26.89.

*3,6-Dimethyl-1-phenyl-5-phenylamino-1,5-dihydropyrazolo[3,4-d]pyrimidin-4-one* (**7**). A mixture of 3,6-dimethyl-1-phenyl-1*H*-pyrazolo[3,4-*d*][1,3]oxazin-4-one (**3**, 2.41 g, 10 mmol) and phenylhydrazine (1.08 gm, 10 mmol) in ethanol (20 mL) was heated under reflux for 6 h. The separated solid was filtered while hot, dried and crystallized from butanol. Mp 265–266 °C; yield: 54%; IR (cm^−1^): 3250 (NH); 3115–3011 (CH aromatic); 2930 (CH aliphatic); 1685 (C=O); ^1^H-NMR (DMSO-*d_6_*) *δ* ppm: 2.49 (s, 3H); 2.53 (s, 3H); 6.69 (d, *J* = 7.2 Hz, 2H); 6.86 (t, *J* = 7.5 Hz, 1H); 7.21 (t, *J* = 7.5 Hz, 2H); 7.39 (t, *J* = 7.8 Hz, 1H); 7.56 (t, *J* = 7.8 Hz, 2H); 8.05 (d, *J* = 9 Hz, 2H); 9.09 (s, 1H); MS *m/z*: 331 (M^+^, 100); Anal. Calcd for C_19_H_17_N_5_O (331.38): C, 68.87; H, 5.17; N, 21.13. Found: C68.68; H, 5.09; N, 21.05.

#### 3.1.2. General Procedure for the Synthesis of 3,6-Dimethyl-1-phenyl-5-(4-substitutedphenyl)-1,5-dihydropyrazolo[3,4-*d*]pyrimidin-4-ones **8a**–**e**

A mixture of 3,6-dimethyl-1-phenyl-1*H*-pyrazolo[3,4-*d*][1,3]oxazin-4-one (**3**, 2.41 g, 10 mmol) and the appropriate aromatic amine (10 mmol) in dry pyridine (30 mL) was heated under reflux for 6 h. The reaction mixture was poured into ice-cold water and the mixture was neutralized with hydrochloric acid (10%). The separated product was filtered, washed with water, dried and crystallized from the appropriate solvent.

*3,6-Dimethyl-1-phenyl-5-p-tolyl-1,5-dihydropyrazolo[3,4-d]pyrimidin-4-one* (**8a**). Mp 170–171 °C (crystallized from methanol); yield: 50%; IR (cm^−1^): 2923 (CH aliphatic); 1693 (C=O); 1568 (C=N); ^1^H-NMR (DMSO-*d_6_*) *δ* ppm: 2.16 (s, 3H); 2.51 (s, 3H); 2.66 (s, 3H); 7.38–7.41 (m, 1H); 7.56–7.62 (m, 4H); 8.07–8.15 (m, 4H); MS *m/z*: 330 (M^+^, 100); Anal. Calcd for C_20_H_18_N_4_O (330.39): C, 72.71; H, 5.49; N, 16.96. Found: C, 72.77; H, 5.20; N, 16.64.

*3,6-Dimethyl-5-(4-hydroxyphenyl)-1-phenyl-1,5-dihydropyrazolo[3,4-d]pyrimidin-4-one* (**8b**). Mp 250–252 °C (crystallized from acetone); yield: 76%; IR (cm^−1^): 3439 (OH); 2930 (CH aliphatic); 1694 (C=O); 1566 (C=N); ^1^H-NMR (DMSO-*d_6_*) *δ* ppm: 2.17 (s, 3H); 2.51 (s, 3H); 6.89 (d, *J* = 9 Hz, 2H); 7.17 (d, *J* = 9 Hz, 2H); 7.38 (t, *J* = 7.5 Hz, 1H); 7.53 (t, *J* = 7.5 Hz, 1H); 8.07 (d, *J* = 9 Hz, 2H); 9.80 (s, IH); MS *m/z*: 332 (M^+^, 100); Anal. Calcd for C_19_H_16_N_4_O_2_ (332.36): C, 68.66; H, 4.85; N, 16.86. Found: C, 68.30; H, 4.50; N, 16.98.

*5-(4-Acetylphenyl)-3,6-dimethyl-1-phenyl-1,5-dihydropyrazolo[3,4-d]pyrimidin-4-one* (**8c**). Mp 186–187 °C (crystallized from ethanol); yield: 66%; IR (cm^−1^): 2924 (CH aliphatic); 1699 (broad band of 2C=O); 1591 (C=N); ^1^H-NMR (DMSO-*d_6_*) *δ* ppm: 2.17 (s, 3H); 2.44 (s, 3H); 2.50 (s, 3H); 7.23 (d, *J* = 9 Hz, 2H); 7.26 (d, *J* = 9 Hz, 2H); 7.52–7.64 (m, 3H); 8.08 (d, *J* = 9 Hz, 2H); MS *m/z*: 358 (M^+^, 100); Anal. Calcd for C_21_H_18_N_4_O_2_ (358.40): C, 70.38; H, 5.06; N, 15.63. Found: C, 69.97; H, 4.98; N, 15.94.

*4-(3,6-Dimethyl-4-oxo-1-phenyl-1,5-dihydropyrazolo[3,4-d]pyrimidin-5-yl)benzoic acid* (**8d**). Mp 200–201 °C (crystallized from acetic acid); yield: 59%; IR (cm^−1^): 3450 (OH); 3068 (CH aromatic); 2929 (CH aliphatic); 1713, 1678 (2C = O); 1558 (C = N); ^1^H NMR (DMSO-*d_6_*) *δ* ppm: 2.16 (s, 3H); 2.51 (s, 3H); 7.38 (t, *J* = 7.2 Hz, 1H); 7.54-7.65 (m, 4H); 8.07-8.13 (m, 4H); 13.15 (s, IH); MS *m/z*: 360 (M^+^, 79.41); Anal. Calcd for C_20_H_16_N_4_O_3_ (360.38): C, 66.66; H, 4.48; N, 15.55. Found: C, 66.90; H, 4.71; N, 15.78.

*Ethyl 4-(3,6-dimethyl-4-oxo-1-phenyl-1,5-dihydropyrazolo[3,4-d]pyrimidin-5-yl)benzoate* (**8e**). Mp 190–191 °C (crystallized from ethanol); yield: 85%; IR (cm^−1^): 2925 (CH aliphatic); 1709 (broad band of 2C=O); 1555 (C=N); ^1^H NMR (DMSO-*d_6_*) *δ* ppm: 1.36 (t, *J* = 7.2 Hz, 3H); 2.15 (s, 3H); 2.51 (s, 3H); 4.37 (q, *J* = 7.2 Hz, 2H); 7.39 (t, *J* = 7.2 Hz, 1H); 7.53–7.62 (m, 4H); 8.07–8.15 (m, 4H); MS *m/z*: 388 (M^+^, 93.03); Anal. Calcd for C_22_H_20_N_4_O_3_ (388.43): C, 68.03; H, 5.19; N, 14.42. Found: C 68.21; H, 5.51; N, 14.66.

*5-Amino-3,6-dimethyl-1-phenyl-1,5-dihydropyrazolo[3,4-d]pyrimidin-4-one* (**9**). A mixture of compound **3** (2.41 g, 10 mmol) and hydrazine hydrate (0.5 mL, 10 mmol) in butanol (20 mL) was heated under reflux for 6 h. After cooling, the separated solid was filtered, dried and crystallized from ethanol. Mp 129–130 °C; yield: 47%; IR (cm^−1^): 3322–3262 (forked, NH_2_); 3065 (CH aromatic); 2924 (CH aliphatic); 1692 (C=O); ^1^H-NMR (DMSO-*d_6_*) *δ* ppm: 2.50 (s, 3H); 2.59 (s, 3H); 5.71 (s, 2H); 7.34 (t, *J* = 7.5 Hz, 1H); 7.52 (t, *J* = 7.5 Hz, 2H); 8.05 (d, *J* = 9 Hz, 2H); MS *m/z*: 255 (M^+^, 100). Anal. Calcd for C_13_H_13_N_5_O (255.28): C, 61.17; H, 5.13; N, 27.43. Found: C, 61.53; H, 5.03; N, 27.23.

#### 3.1.3. General Procedure for the Synthesis of (*E*)-3,6-Dimethyl-5-(4-substitutedbenzylideneamino)-1-phenyl-1,5-dihydropyrazolo[3,4-*d*]pyrimidin-4-ones **10a**–**e**

A mixture of compound **9** (2.55 gm, 10 mmol) and the appropriate aromatic aldehyde (12 mmol) in acetic acid (15 mL) was heated under reflux for 20 h. The reaction mixture was concentrated under vacuum then cooled. The obtained solid was filtered, washed with water, dried and crystallized from the appropriate solvent. The spectral data for compounds **10a**–**e** are listed below.

*(E)-5-(Benzylideneamino)-3,6-dimethyl-1-phenyl-1,5-dihydropyrazolo[3,4-d]pyrimidin-4-one* (**10a**). Mp 250–251 °C (crystallized from ethanol); yield: 70%; IR (cm^−1^): 3055 (CH aromatic); 2925 (CH aliphatic); 1702 (C=O); 1555 (C=N); ^1^H-NMR (DMSO-*d_6_*) *δ* ppm: 2.50 (s, 3H); 2.55 (s, 3H); 7.39 (t, *J* = 9 Hz, 1H); 7.53-7.65 (m, 5H); 7.98 (d, *J* = 9Hz, 2H); 8.08 (d, *J* = 9 Hz, 2H); 8.89 (s, 1H); MS *m/z*: 343 (M^+^, 4.62); Anal. Calcd for C_20_H_17_N_5_O (343.39): C, 69.96; H, 4.99; N, 20.39. Found: C, 69.77; H, 5.20; N, 20.54.

*(E)-3,6-Dimethyl-5-(4-fluorobenzylideneamino)-1-phenyl-1,5-dihydropyrazolo[3,4-d]pyrimidin-4-one* (**10b**). Mp 160–161 °C (crystallized from methanol); yield: 79%; IR (cm^−1^): 3067 (CH aromatic); 2928 (CH aliphatic); 1709 (C=O); 1596 (C=N); ^1^H NMR (DMSO-*d_6_*) *δ* ppm: 2.10 (s, 3H); 2.41 (s, 3H); 7.38–7.40 (m, 1H); 7.52–7.57 (m, 4H); 7.99-8.01 (m, 4H); 10.94 (s, 1H); Anal. Calcd for C_20_H_16_FN_5_O (361.38): C, 66.47; H, 4.46; N, 19.38. Found: C, 66.38; H, 4.02; N, 19.61.

*(E)-3,6-Dimethyl-5-(4-methoxybenzylideneamino)-1-phenyl-1,5-dihydropyrazolo[3,4-d]pyrimidin-4-one* (**10c**). Mp 255–256 °C (crystallized from toluene); yield: 55%; IR (cm^−1^): 3027 (CH aromatic); 1671 (C=O); 1594 (C=N); ^1^H-NMR (DMSO-*d_6_*) *δ* ppm: 2.49 (s, 3H); 2.51 (s, 3H); 3.84 (s, 3H); 7.22 (t, *J* = 6 Hz, 1H); 7.37–7.57 (m, 6H); 8.07 (d, *J* = 9Hz, 2H); 8.85 (s, 1H); MS *m/z*: 373 (M^+^, 10.42); Anal. Calcd for C_21_H_19_N_5_O_2_ (373.42): C, 67.55; H, 5.13; N, 18.75. Found: C, 67.10; H, 5.01; N, 18.47.

*(E)-5-(4-Chlorobenzylideneamino)-3,6-dimethyl-1-phenyl-1,5-dihydropyrazolo[3,4-d]pyrimidin-4-one* (**10d**). Mp > 300 °C (crystallized from ethanol); yield: 60%; IR (cm^−1^): 3064 (CH aromatic); 2925 (CH aliphatic); 1698 (C=O); 1596 (C=N); ^1^H-NMR (DMSO-*d_6_*) *δ* ppm: 2.09 (s, 3H); 2.54 (s, 3H); 7.37 (t, *J* = 8.1 Hz); 7.56 (t, *J* = 8.1 Hz, 2H); 7.66 (d, *J* = 8.4 Hz, 2H); 7.99 (d, *J* = 8.4 Hz, 2H); 8.32 (d, *J* = 8.7 Hz, 2H); 8.32 (s, 1H); ^13^C-NMR (DMSO-*d_6_*) *δ* ppm: 13.72, 23.32, 104.40, 121.72, 127.13, 129.68, 129.83, 130.93, 131.63, 137.96, 138.66, 146.86, 150.30, 154.86, 157.74, 168.91. MS *m/z*: 377 (M^+^, 11.12), Anal. Calcd for C_20_H_16_ClN_5_O (377.85): C, 63.58; H, 4.27; N, 18.51. Found: C, 63.28; H, 4.04; N, 18.13.

*(E)-3,6-Dimethyl-5-(4-nitrobenzylideneamino)-1-phenyl-1,5-dihydropyrazolo[3,4-d]pyrimidin-4-one* (**10e**). (Crystallized from acetic acid); mp > 300 °C; yield: 95%; IR (cm^−1^): 3057 (CH aromatic); 2925 (CH aliphatic); 1690 (C=O); 1597 (C=N); ^1^H-NMR (DMSO-*d_6_*) *δ* ppm: 2.60 (s, 3H); 2.61 (s, 3H); 7.39 (t, *J* = 7.8 Hz, 1H); 7.57 (t, *J* = 7.8 Hz, 2H); 8.07 (d, *J* = 8.7 Hz, 2H); 8.25 (d, *J* = 9 Hz, 2H); 8.42 (d, *J* = 9 Hz, 2H); 9.16 (s, 1H); MS *m/z*: 388 (M^+^, 18.10). Anal. Calcd for C_20_H_16_N_6_O_3_ (388.39): C, 61.85; H, 4.15; N, 21.64. Found: C, 62.15; H, 4.44; N, 21.40.

### 3.2. Biological Testing

#### 3.2.1. Materials and Methods

Human breast cancer cell line, MCF was grown as monolayer culture in RPM 11640 medium supplemented with 10% fetal bovine serum (FBS) and 1% penicillin/streptomycin. The cell line was incubated at 37 °C 5% CO_2_ 95% air and high humidity atmosphere in the water jacketed incubator (Revco, GS Laboratory Equipment, RCO 3000 TVBB, Asheville, NC, USA). The cell line was regularly subcultured to be maintained in the exponential growth phase. The sterile conditions were strictly attained by working under the equipped laminar flow (Microflow laminar flow cabinet, Hamsphire SP 105aa, Andover, UK).

#### 3.2.2. Measutement of Potential Cytotoxicity

The cytotoxicity was carried out using the sulphorhodamine-B (SRB) assay. Cells were seeded in 96 well microtiter plates at a concentration of 1,000–2,000 cells/well, 100 µL/well, After 24 h, cells will be incubated for 72 h with various concentrations of drugs (0, 0.01, 0.1, 1, 10 and 100 µg/mL). For each derivative concentration and doxorubicin, 3 wells were used. The plates were incubated for 72 h. The medium is discarded. The cells were fixed with 150 μL cold trichloroacetic acid 10% final concentration for 1 h at 4 °C.

The plates were washed with distilled water using a Tecan automatic washer (Crailsheim, Germany) and stained with 50 μL 0.4% SRB dissolved in 1% acetic acid for 30 min at room temperature in dark. The plates were washed with 1% acetic acid to remove unbound dye and air-dried (24 h).

The dye was solubilized with 150 µL/well of 10 mMtris base (PH 7.4) for 5 min on a shaker at 1,600 rpm. The optical density (OD) of each well will be measured spectrophotometrically at 490 nm with an ELISA microplate reader. The mean background absorbance was automatically subtracted and mean values of each derivative and doxorubicin concentration was calculated. The experiment was repeated 3 times. The percentage of cell survival was calculated as follows:

Surviving fraction = O.D. (treated cells)/O.D. (control cells)

## 4. Conclusions

In conclusion, series of new 5-substituted pyrazolo[3,4-*d*]pyrimidin-4-ones **4**, **5a**,**b**, **6**, **7**, **8a**–**e**, **10a**–**e** were synthesized. The cytotoxic activity of the newly synthesized compounds against the human breast adenocarcinoma cell line MCF-7 was investigated. Almost all the tested compounds revealed some antitumor activity, in particular 3,6-dimethyl-5-(4-nitrobenzylideneamino)-1-phenyl-1,5-dihydropyrazolo[3,4-*d*]pyrimidin-4-one (**10e**) which exhibited the highest activity among the tested compounds with an IC_50_ equal to 11 µM. The antitumor screening revealed that aromatic substitution on the N5 position favors the activity. Moreover, 5-(4-substituted benzylideneamino) derivatives **10a**–**e** with azomethine spacer groups were more potent than the 5-(4-substituted phenyl) derivatives **8a**–**e**.

## References

[1] Wu H., Chang D., Huang C. (2006). Targeted therapy for cancer. J. Cancer Mol..

[2] Sierra J.R., Cepero V., Giordano S. (2010). Molecular mechanisms of acquired resistance to tyrosine kinase targeted therapy. Mol. Cancer.

[3] Riley L.B., Desai D.C. (2009). The molecular basis of cancer and the development of targeted therapy. Surg. Clin. N. Am..

[4] Cheng C.C., Robins R.K. (1956). Potential Purine Antagonists. VI. Synthesis of 1-alkyl- and 1-aryl-4-substituted pyrazolo[3,4-d]pyrimidines. J. Org. Chem..

[5] Ismail Z.H., Abdel-Gawad S.M., Abdel-Aziem A., Ghorab M.M. (2003). Synthesis of some new biologically active sulfur compounds containing pyrazolo[3,4-d]pyrimidine moiety. Phosphor. Sulfur Silicon.

[6] Carraro F., Naldini A., Pucci A., Locatelli G.A., Maga G., Schenone S., Bruno O., Ranise A., Bondavalli F., Brullo C. (2006). Pyrazolo[3,4-d]pyrimidines as potent antiproliferative and proapoptotic agents toward A431 and 8701-BC cells in culture via inhibition of c-Src phosphorylation. J. Med. Chem..

[7] El-Enany M.M., Kamel M.M., Khalil O.M., El-Nassan H.B. (2010). Synthesis and antitumor activity of novel 6-aryl and 6-alkylpyrazolo[3,4-d]pyrimidin-4-one derivatives. Eur. J. Med. Chem..

[8] Abd El Hamid M.K., Mihovilovic M.D., El-Nassan H.B. (2012). Synthesis of novel pyrazolo[3,4-d]pyrimidine derivatives as potential anti-breast cancer agents. Eur. J. Med. Chem..

[9] Kandeel M.M., Mohamed L.W., Abd El Hamid M.K., Negmeldin A.T. (2012). Design, synthesis, and antitumor evaluation of novel pyrazolo[3,4-d]pyrimidine derivatives. Sci. Pharm..

[10] Peat A.J., Garrido D., Boucheron J.A., Schweiker S.L., Dickerson S.H., Wilson J.R., Wang T.Y., Thomson S.A. (2004). Novel GSK-3 inhibitors with improved cellular activity. Bioorg. Med. Chem. Lett..

[11] Kim D.C., Lee Y.R., Yang B.S., Shin K.J., Kim D.J., Chung B.Y., Yoo K.H. (2003). Synthesis and biological evaluations of pyrazolo[3,4-d]pyrimidines as cyclin-dependent kinase 2 inhibitors. Eur. J. Med. Chem..

[12] Schenone S., Brullo C., Bruno O., Bondavalli F., Mosti L., Maga G., Crespan E., Carraro F., Manetti F., Tintori C. (2008). Synthesis, biological evaluation and docking studies of 4-amino substituted 1H-pyrazolo[3,4-d]pyrimidines. Eur. J. Med. Chem..

[13] Schenone S., Bruno O., Bondavalli F., Ranise A., Mosti L., Menozzi G., Fossa P., Manetti F., Morbidelli L., Trincavelli L. (2004). 1-(2-Chloro-2-phenylethyl)6-methylthio-1H-pyrazolo[3,4-d]pyrimidines-4-amino substituted and their biological evaluation. Eur. J. Med. Chem..

[14] Ghorab M.M., Ragab F.A., Alqasoumi S.I., Alafeefy A.M., Aboulmagd S.A. (2010). Synthesis of some new pyrazolo[3,4-d]pyrimidine derivatives of expected anticancer and radioprotective activity. Eur. J. Med. Chem..

[15] Hassan G.S., Kadry H.H., Abou-Seri S.M., Ali M.M., Mahmoud A.E. (2011). Synthesis and *in vitro* cytotoxic activity of novel pyrazolo[3,4-d]pyrimidines and related pyrazole hydrazones toward breast adenocarcinoma MCF-7 cell line. Bioorg. Med. Chem..

[16] Reck M., Mok T., Wolf J., Heigener D., Wu Y.L. (2011). Reviewing the safety of erlotinib in non small cell lung cancer. Expert Opin. Drug Saf..

[17] Omar H.A., Sargeant A.M., Weng J.-R., Wang D., Kulp S.K., Patel T., Chen C.-S. (2011). Pulmonary endothelial impairment during gefitinib therapy: A preliminary assessment with iodine-123-metaiodobenzylguanidine (1231-MIBG) scintigraphy. Open Lung Cancer J..

[18] Fang L., Barekati Z., Zhang B., Liu Z., Zhong X. (2011). Targeted therapy in breast cancer: What’s new?. Eur. J. Med. Sci..

[19] Bakr R.B., Abdelall E.K.A., Abdel-Hamid M.K., Kandeel M.M. (2012). Design and synthesis of new EGFR-tyrosine kinase inhibitors containing pyrazolo[3,4-d]pyrimidine cores as anticancer agents. Bull. Pharm. Sci. Assiut Univ..

[20] Heravi M.M., Nami N., Seifi N., Oskooie H.A., Hekmatshoar R. (2006). Microwave-assisted synthesis of substituted pyrazoles and pyrazolo[3,4-d]thiopyrimidines. Phosphor. Sulfur Silicon.

[21] Vichai V., Kirtikara K. (2006). Sulforhodamine B colorimetric assay for cytotoxicity screening. Nat. Protoc..

